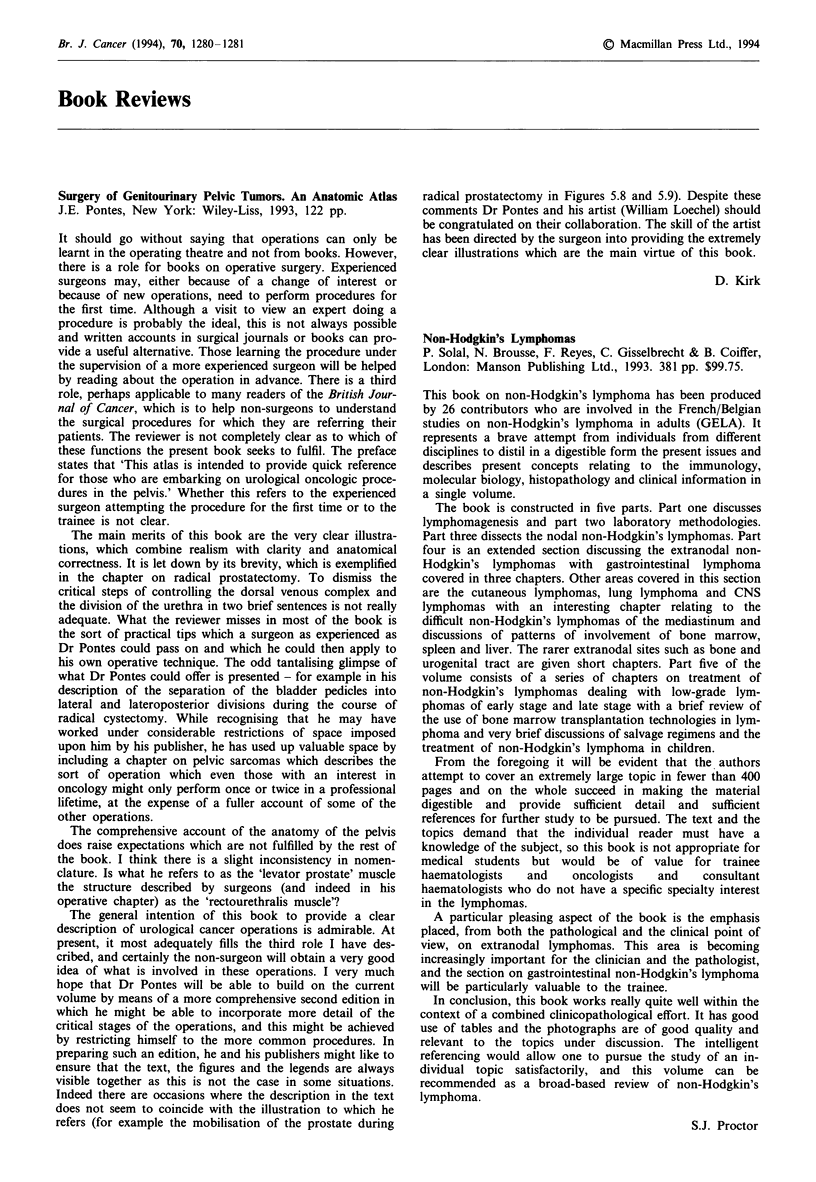# Non-Hodgkin's Lymphomas

**Published:** 1994-12

**Authors:** S.J. Proctor


**Non-Hodgkin's Lymphomas**
P. Solal, N. Brousse, F. Reyes, C. Gisselbrecht & B. Coiffer, London: Manson Publishing Ltd., 1993. 381 pp. $99.75.

This book on non-Hodgkin's lymphoma has been produced by 26 contributors who are involved in the French/Belgian studies on non-Hodgkin's lymphoma in adults (GELA). It represents a brave attempt from individuals from different disciplines to distil in a digestible form the present issues and describes present concepts relating to the immunology, molecular biology, histopathology and clinical information in a single volume.

The book is constructed in five parts. Part one discusses lymphomagenesis and part two laboratory methodologies. Part three dissects the nodal non-Hodgkin's lymphomas. Part four is an extended section discussing the extranodal non-Hodgkin's lymphomas with gastrointestinal lymphoma covered in three chapters. Other areas covered in this section are the cutaneous lymphomas, lung lymphoma and CNS lymphomas with an interesting chapter relating to the difficult non-Hodgkin's lymphomas of the mediastinum and discussions of patterns of involvement of bone marrow, spleen and liver. The rarer extranodal sites such as bone and urogenital tract are given short chapters. Part five of the volume consists of a series of chapters on treatment of non-Hodgkin's lymphomas dealing with low-grade lymphomas of early stage and late stage with a brief review of the use of bone marrow transplantation technologies in lymphoma and very brief discussions of salvage regimens and the treatment of non-Hodgkin's lymphoma in children.

From the foregoing it will be evident that the authors attempt to cover an extremely large topic in fewer than 400 pages and on the whole succeed in making the material digestible and provide sufficient detail and sufficient references for further study to be pursued. The text and the topics demand that the individual reader must have a knowledge of the subject, so this book is not appropriate for medical students but would be of value for trainee haematologists and oncologists and consultant haematologists who do not have a specific specialty interest in the lymphomas.

A particular pleasing aspect of the book is the emphasis placed, from both the pathological and the clinical point of view, on extranodal lymphomas. This area is becoming increasingly important for the clinician and the pathologist, and the section on gastrointestinal non-Hodgkin's lymphoma will be particularly valuable to the trainee.

In conclusion, this book works really quite well within the context of a combined clinicopathological effort. It has good use of tables and the photographs are of good quality and relevant to the topics under discussion. The intelligent referencing would allow one to pursue the study of an individual topic satisfactorily, and this volume can be recommended as a broad-based review of non-Hodgkin's lymphoma.

S.J. Proctor